# Integrated machine learning analysis of 30 cell death patterns identifies a novel prognostic signature in glioma

**DOI:** 10.3389/fcell.2025.1677290

**Published:** 2025-09-19

**Authors:** Minhao Huang, Kai Zhao, Yongtao Yang, Kexin Mao, Hangyu Ma, Tingting Wu, Guolin Shi, Wenhu Li, Yan Li, Ruiqi Peng, Ying Cheng, Ninghui Zhao

**Affiliations:** ^1^ Department of Neurosurgery, The Second Affiliated Hospital of Kunming Medical University, Kunming, China; ^2^ Institute of Biomedical Research, Yunnan University, Kunming, China; ^3^ Southwest United Graduate School, Kunming, China

**Keywords:** glioma, programmed cell death, machine learning, immune microenvironment, drug sensitivity, prognostic model

## Abstract

**Background:**

Glioma heterogeneity and therapeutic resistance are closely linked to dysregulated programmed cell death (PCD). While individual PCD pathways have been studied, the integrated network of multi-modal PCD interactions and their clinical implications in glioma remain poorly understood. This study aims to decipher the interplay between 30 distinct PCD modalities and the immune microenvironment, developing a robust prognostic signature to guide therapy.

**Methods:**

This study integrated 2,743 glioma samples from TCGA, CGGA, and GEO databases, encompassing RNA-seq, single-cell transcriptomic (GSE167960), and mutational data. Through literature mining and GeneCards database screening, 30 programmed cell death (PCD)-related gene sets (total 11,681 genes) were curated, identifying 428 differentially expressed genes (DEGs; |log_2_FC|>1, p < 0.05). A pan-death prognostic signature (Cell-Death Score, CDS) was constructed using 114 machine learning algorithm combinations, refined via CoxBoost to select 25 key genes. CIBERSORT quantified the abundance of 22 immune cell subsets, while ssGSEA assessed functional activity of 28 immune cell types. Drug sensitivity predictions employed GDSC database, with single-cell trajectory analysis validating molecular mechanisms and therapeutic strategies. *In vitro*, differential expression profiles of key genes were first examined between human normal astrocyte cell lines (SVG-P12) and three glioma cell lines (U87, U251, LN229). Subsequently, RNA-seq and qRT-PCR validated expression patterns of 25 key genes in tumor/adjacent non-tumorous tissues from 7 glioma patients. Finally, spatial transcriptomic data from 4 glioma tissue samples in our cohort (including two paired tumor-adjacent non-tumorous samples and two tumor-only samples) were integrated to delineate spatial expression patterns of key genes.

**Results:**

Integrated analysis of 2,743 public gliomas samples identified 428 cell death-associated differentially expressed genes, enriched in neuroactive ligand-receptor interactions and extracellular matrix regulation. Unsupervised clustering revealed distinct immune-activated and immune-silent patient subtypes. A pan-death prognostic signature (Cell-Death Score, CDS), constructed via multi-algorithm machine learning and optimized using CoxBoost to incorporate 25 key genes, demonstrated robust performance in training (1-/3-year AUC = 0.894/0.943) and validation cohort (C-index = 0.717), effectively stratifying high-risk patients (HR = 3.21, p < 0.0001). High-CDS patients displayed elevated tumor mutational burden, homologous recombination deficiency, and immune checkpoint expression, alongside enhanced sensitivity to 11 therapeutic agents, including gemcitabine. Single-cell trajectory analysis confirmed significant activation of model genes during glioma progression. A clinical nomogram integrating CDS, WHO grade and radiotherapy further improved prognostic utility. Based on *in vitro* cell line experiments, the expression profiles of 25 key genes demonstrated significant heterogeneity, with partial genes undetectable by qRT-PCR due to expression levels falling below detection thresholds. Among seven genes consistently detected across all 4 cell lines, tumor cell lines exhibited significantly upregulated expression relative to normal astrocyte counterparts. RNA-seq analysis revealed effective detection of 24/25 key genes in seven paired tumor/adjacent tissue samples, with 20 genes showing higher mean expression in tumor tissues. qRT-PCR validation confirmed upregulated trends for 12 detectable genes in tumor tissues. Spatial transcriptomic analysis further corroborated tumor region-specific overexpression of all 25 key genes compared to adjacent non-tumorous areas.

**Conclusion:**

The CDS signature unravels the molecular interplay between glioma cell death heterogeneity, immune dysregulation, and therapeutic resistance. This biomarker system provides both prognostic and therapeutic insights for precision oncology, paving the way for personalized combination therapies in glioma management.

## Introduction

Gliomas represent the most prevalent primary tumors of the human central nervous system (CNS), with current evidence suggesting their origin in neural stem or progenitor cells (Yang et al., 2022). According to the latest WHO classification of CNS tumors, gliomas are classified from WHO 1 to 4 based on malignancy, where glioblastoma constitutes the most aggressive subtype (Louis et al., 2021). Surgical resection remains the primary therapeutic intervention; nevertheless, complete resection is often unattainable due to tumors’ invasive growth patterns and anatomical integration with adjacent tissues (Nabors et al., 2020). Moreover, despite multimodal therapy combining surgery, radiotherapy and chemotherapy, patient prognosis persists as unfavorable, driven by high tumor heterogeneity, an immunologically suppressive (“cold”) tumor microenvironment (TME), and the infiltrative capacity of glioma stem cells (Weller et al., 2024; Liu et al., 2024a). Uncontrolled proliferation defines gliomas pathobiology, promoting increased focus on regulatory role of tumor cell death in disease progression (Mancusi and Monje, 2023). While current research on diagnostic biomarkers and therapeutic agents for glioma has made progress (Ivo D'Urso et al., 2015; Bombino et al., 2024), studies integrating 30 cell death modalities to address this issue remain largely unexplored. Consequently, elucidating the impact of diverse cell death modalities on gliomagenesis, establishing reliable prognostic models, and identifying molecular therapeutic targets promise new foundational insight for glioma treatment.

Cellular death predominantly arises via two pathways: accident cell death (ACD) (Tang et al., 2019) and programmed cell death (PCD) (Galluzzi et al., 2018). ACD is an uncontrolled biological process, whereas PCD is a tightly regulated biological process involving multiple molecular pathways and mechanisms critical for maintaining cellular homeostasis and eliminating abnormal cells (Galluzzi et al., 2018; Qin et al., 2023). PCD manifests in diverse forms, including apoptosis, necroptosis, ferroptosis, cuproptosis, pyroptosis, alkaliptosis, lysosome-dependent death, and autophagy-dependent death (Hanahan and Weinberg, 2011). Comprehensive literature and GeneCards database analyses currently recognize 30 distinct PCD modalities. Increasing evidence demonstrates that PCD fundamentally influences malignant tumor progression, as cancer cells evade multiple PCD forms during tumorigenesis (Su et al., 2015). Dysregulated PCD is closely associated with key malignant phenotypes, including tumor proliferation, metastasis, and recurrence (Yu et al., 2021; Yan et al., 2022), with numerous studies confirming a strong link between glioma progression and PCD (Hanson et al., 2023; Wei et al., 2024). However, the molecular characteristics of PCD in gliomas and its clinical therapeutic potential remain insufficiently understood, necessitating further exploration into PCD-glioma cross-talk to advance treatment strategies.

During glioma progression, tumor cells selectively recruit immunosuppressive cell populations to establish an immune-suppressive microenvironment, a pathological mechanism identified as a major cause of immunotherapy failure (Quai et al., 2017). Simultaneously, PCD activation triggers release of inflammatory cytokines, chemokines, and immunoregulatory molecules (Dai et al., 2020; Park and Chung, 2019; Liu et al., 2022). To address these complexities, this study integrated data from 2,743 glioma patients across TCGA, CGGA, and GEO databases. We systematically analyzed 30 cell death modalities and constructed a pan-death prognostic signature (Cell-Death Score, CDS) using 114 machine learning algorithm combinations. Employing advanced bioinformatics, we identified 25 key genes, deciphered interaction linking PCD modalities to the immune microenvironment, and validated candidate therapeutic agents. These findings provide novel insights into the role of PCD in glioma progression and contribute to the development of improved therapeutic approaches.

## Results

### Genetic characteristics associated with cell death are enriched in the ligand-receptor interaction pathways

**FIGURE 1:**
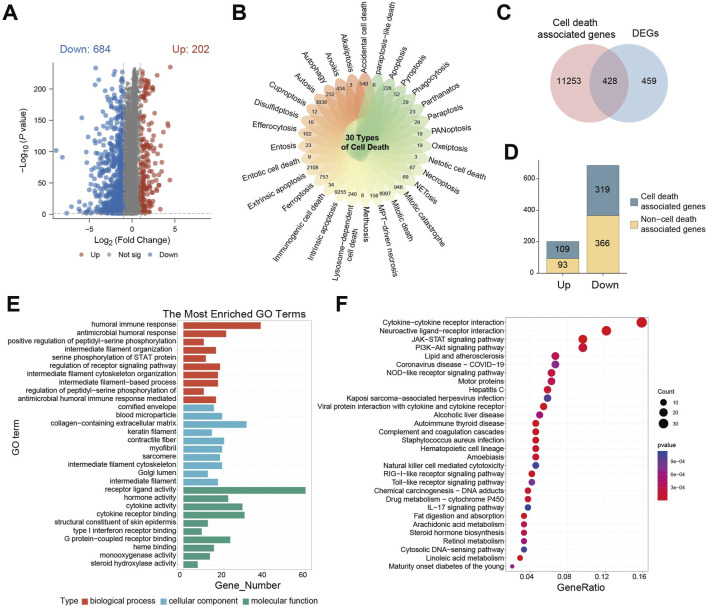
Genetic characteristics associated with cell death are enriched in the ligand-receptor interaction pathways. **(A)** Differential gene expression volcano plot between GTEx cohort and TCGA- GBM/LGG cohort; **(B)** petal plot of the number of related genes corresponding to 30 cell death modes; **(C)** Venn diagram of intersection of cell death-related genes and differential genes; **(D)** Bar chart of the number of cell death-related genes in upregulated/downregulated differential genes; **(E)** GO enrichment analysis of cell death-related differential gene enrichment pathway bar chart; **(F)** KEGG enrichment analysis of cell death-related differential gene enrichment pathway bubble map, the color of the bubble indicates the P value of enrichment significance, and the size of the bubble indicates the number of enriched genes.

To explore differences in 30 cell death-related genes between normal brain tissues and gliomas patients, we analyzed gene expression profiles from GTEx cohort (normal brain) and TCGA-GBM/LGG cohort (glioma). This analysis identified 886 statistically differentially expressed genes (DEGs), including 202 upregulated and 684 downregulated genes (Figure 1A). Visualization using a petal plot revealed the number of genes associated with each of the 30 cell death modes, ranging from 3 to 9,255 (Figure 1B). Integrating all cell death-related genes yielded a total of 11,681 genes. Intersection of these with the 886 DEGs identified 428 cell death-associated DEGs (Figure 1C). Among these, 109 were upregulated and 319 were downregulated (Figure 1D).

Gene Ontology (GO) enrichment analysis of these 428 DEGs revealed significant associations. Within biological processes, the humoral immune response was the most enriched term. This adaptive immune process, involving B cell-mediated antibody production, functions in concert with cell-mediated immunity driven by T cells. For cellular components, the term “extracellular matrix containing collagen” was most enriched, highlighting the structural and functional importance of collagen-rich matrices, which are implicated in tumor cell invasion, metastasis, and microenvironment regulation. In terms of molecular function, receptor-ligand activity was significantly enriched, reflecting the critical role of receptor-ligand binding in cellular signaling, function, survival, and proliferation (Figure 1E).

Parallel Kyoto Encyclopedia of Genes and Genomes (KEGG) enrichment analysis demonstrated significant enrichment of DEGs in pathways involving cytokine-cytokine receptors interaction, neuroactive ligand-receptor interaction, JAK-STAT signaling, and PI3K−Akt signaling. The significant dysregulation of neuroactive ligand-receptor interactions drives characteristic clinical manifestations in glioma: Tumor cells abnormally secrete neurotransmitters such as glutamate, which not only induce peritumoral epilepsy by activating neuron-associated receptors but also directly accelerate tumor proliferation and metabolic reprogramming through autocrine activation of the mTOR signaling axis. This is closely related to the clinical phenotypes and proliferative features of glioma. Dysregulation of cytokine-receptor pathways mediates sustained recruitment of tumor-associated macrophages, forming an immunosuppressive microenvironment that weakens anti-tumor immune responses. Activation of PI3K-Akt and JAK-STAT pathways leads to broad resistance to radiotherapy, chemotherapy, and targeted therapies by regulating cell cycle progression, inducing anti-apoptotic protein expression, and enhancing DNA damage repair capacity (Figure 1F).

### Identification of cell death-related patient subgroups by unsupervised clustering

**FIGURE 2:**
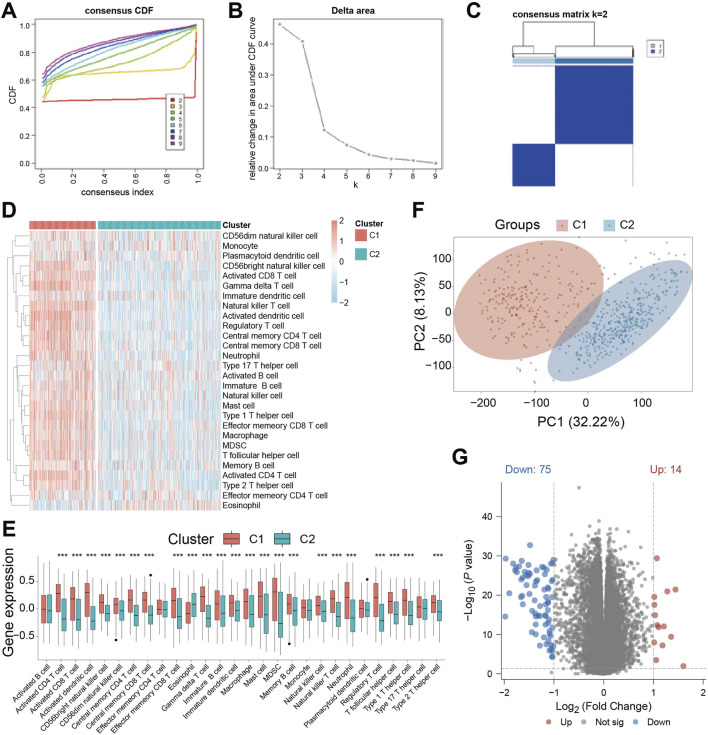
Identification of cell death-related patient subgroups by unsupervised clustering. **(A)** Cumulative Distribution Function (CDF) curves for k = 2-9 in the consistency cluster; **(B)** the relative change curve of the area under the CDF (Cumulative Distribution Function) curve when k = 2-9 in the consistency cluster; **(C)** heat map of consistent clustering (k = 2) of patients in TCGA-GBM/LGG dataset; **(D)** ssGSEA (single - sample Gene Set Enrichment Analysis) immune infiltration Analysis of immune cell abundance differences between C1 and C2 heat map; **(E)** Violin plot of immune cell abundance difference between C1 and C2 in immune infiltration Analysis of ssGSEA (single-sample Gene Set Enrichment Analysis); **(F)** Principal Component Analysis (PCA) cluster plots of C1 and C2 samples; **(G)** Volcano plot of gene expression difference between C1 subgroup and C2 subgroup. *** indicates P < 0.001.

Base on the above-mentioned analyses, we performed unsupervised clustering on the TCGA-GBM/LGG cohort to classify patients based on cell death-related gene expression. Optimal clustering stability was achieved by dividing patients into subgroups C1 and C2 while maximizing intra-group consensus and minimizing ambiguity (Figures 2A–C). Immune infiltration analysis revealed significant differences between subgroups, with C1 exhibiting a generally higher degree of immune cell infiltration (Figure 2D). Specifically, while activated B cells, effector CD4 T cells, monocytes, plasmacytoid dendritic cells, and type 17 helper T cells showed no significant difference, eosinophils abundance was significantly higher in C2. All other immune cell types were significantly more abundant in C1 (Figure 2E). This indicates distinct tumor immune activities between the subgroups defined by cell death patterns. Principal component analysis (PCA) confirmed clear separation between C1 and C2 (Figure 2F), suggesting divergent tumor characteristics. Consequently, we performed differential gene expression analysis between the subgroups, identifying DEGs suing thresholds of P < 0.05, | Log_2_FC | > 1. This analysis yielded 89 significant DEGs: 14 were significantly upregulated, while 75 were significantly downregulated (Figure 2G).

### Prognostic model establishment based on CoxBoost machine learning

**FIGURE 3:**
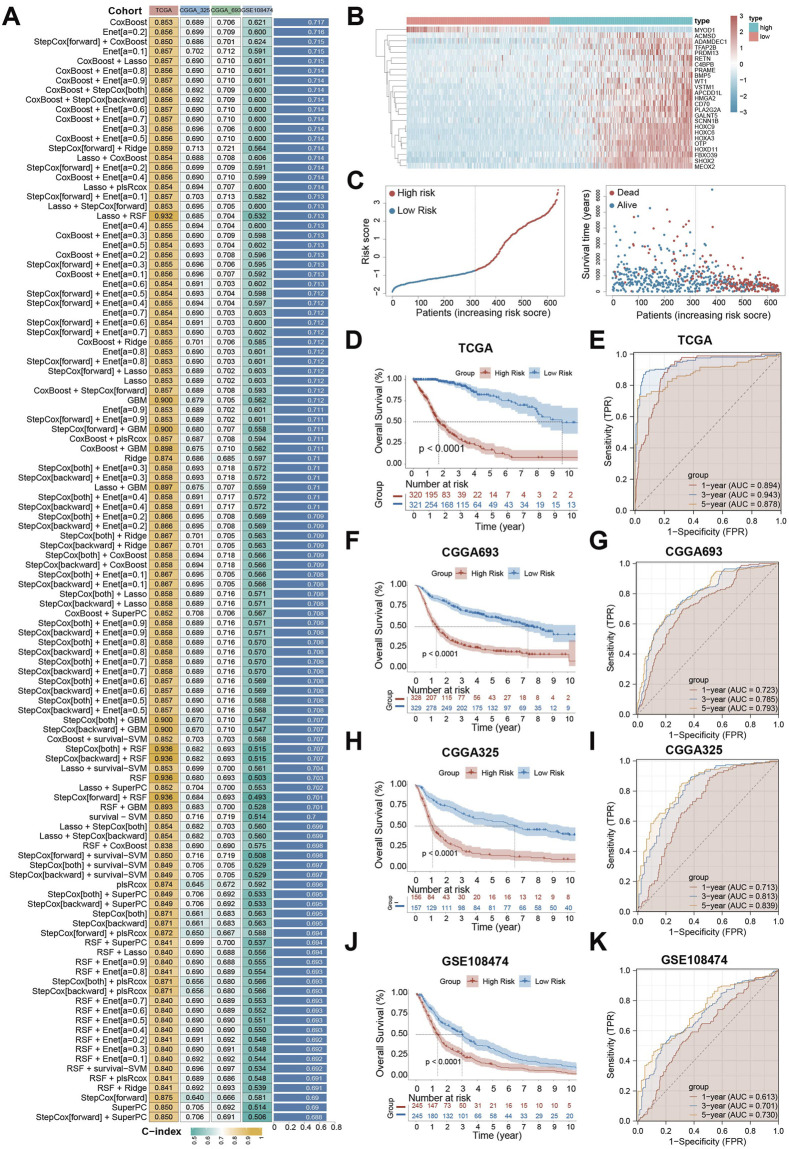
Prognostic model establishment based on CoxBoost machine learning. **(A)** The C-index heat map of 114 machine learning models combined based on 89 key genes in the training set TCGA-GBM/LGG and the validation set CGGA-693, CGGA-325, GSE108474; **(B)** Heat map of differential expression of 25 model genes in CDS between high and low risk groups in TCGA-GBM/LGG; **(C)** Scatter plot of CDS distribution and distribution of high and low score groups in TCGA-GBM/LGG; Scatter plot of survival status distribution of patients ranked by CDS level in TCGA-GBM/LGG; **(D)** KM curves of patients in high and low score groups in TCGA-GBM/LGG; **(E)** receiver operating characteristic (ROC) curve of TCGA-GBM/LGG in high and low risk group; **(F)** KM curve of patients in the high and low risk group of CGGA-693 in the validation set; **(G)** receiver operating characteristic (ROC) curve of CGGA-693 in high and low risk group; **(H)** KM curve of patients in the high and low risk group of validation set CGGA-325; **(I)** validation set CGGA - 325 high risk group of patients with ROC curve; **(J)** KM curve of GSE108474 in the high and low risk group; **(K)** ROC curve of validation set GSE108474 in high and low risk group. Significant dynamic changes were defined as p-value <0.05 and |log_2_FC|>1.

We then focused these 89 key genes and constructed prognosis model using the TCGA-GBM/LGG cohort as the training set, and GSE108474, CGGA-693, and CGGA-325 as validation sets. We evaluated 114 machine learning algorithms. The CoxBoost model was selected as the final prognostic signature due to its superior average C-index (0.717) across all cohorts, along with demonstrated stability in high-dimensional data and consistent performance in cross-validation (Figure 3A). Using this model, we calculated a Cell-Death Score (CDS) for each patient sample. Patients were divided into high- and low-risk groups based on the median CDS value. Expression levels of the 25 genes selected by the CoxBoost prognostic model differed significantly between risk groups, with most genes showing higher expression in the high-risk group (Figure 3B). Distribution of CDS within the TCGA-GBM/LGG cohort is shown in Figure 3C. Dividing patients by increasing CDS revealed a corresponding increase in mortality and decrease in survival time (Figure 3C). Kaplan-Meier (KM) survival analysis was performed on patients in the TCGA- GBM/LGG cohort, and the results showed that patients in the high-risk group had a significantly worse prognosis (P < 0.0001) (Figure 3D). Receiver operating characteristic (ROC) curve analysis demonstrated strong prognostic performance for CDS, with area under the curve (AUC) of 0.894, 0.943 and 0.878 for 1-year, 3-year and 5-year survival, respectively (Figure 3E). Validation in the CGGA-693, CGGA-325, and GSE108474 corhorts consistently showed poorer prognosis for high-risk patients (Figures 3F,H,J), and robust AUC values (mostly >0.7) for 1-, 3-, and 5-year survival prediction (Figures 3G,I,K), confirming CDS as an excellent prognostic model for glioma patients.

### Mutation landscape between CDS high- and low-risk groups exhibits significant differences

**FIGURE 4:**
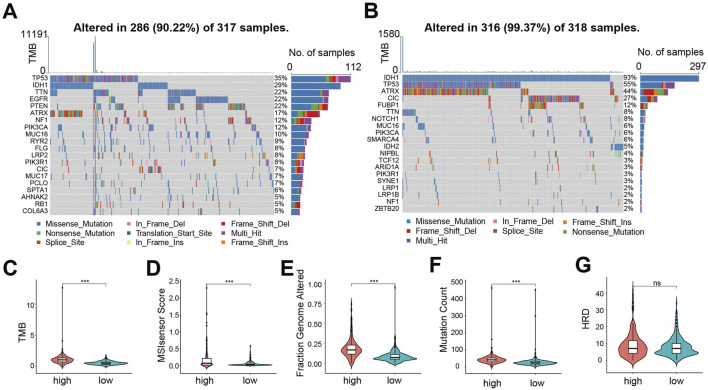
Mutation landscape between CDS high- and low-risk groups exhibits significant differences. **(A)** CDS mutations in patients with high-risk group waterfall figure; **(B)** CDS mutations in patients with low-risk group waterfall figure; **(C)** Violin plot of Tumor Mutation Burden between CDS high and low-risk groups; **(D)** CDS high-risk group of patients with microsatellite instability Score (MSIsensor Score) differences violin figure; **(E)** Violin plot of difference in Fraction Genome Altered between CDS high and low risk groups **(F)** Violin plot of difference in Mutation Count between CDS high and low risk groups; **(G)** Violin plot of Homologous Recombination Deficiency score difference between CDS high and low risk groups. *** indicates P < 0.001, ns indicates no statistical significance.

To explore mutational differences, we analyzed the mutational landscape. In the high-risk group, TP53 mutation was the most frequent (35% of patients), exhibiting diverse mutation types (Figure 4A). Conversely, in the low-risk group, IDH1 mutation predominated (93% of patients), mainly nonsense mutation (Figure 4B). Overall, the proportion of mutations in genes was lower in the low-risk group compared to the high-risk group, except for *IDH1, TP53, ATRX* and *CIC*. Furthermore, we evaluated genomic instability metrics: Tumor Mutation Burden (TMB, Figure 4C), MSIsensor Score (Figure 4D), Fraction Genome Altered (FGA, Figure 4E), Mutation Count (Figure 4F), and Homologous Recombination Deficiency (HRD) score (Figure 4G). All metrics except HRD score were significantly higher in the high-risk group, indicating a higher incidence of gene mutation events and greater genomic instability among these patients.

### Significant differences exist in cell death patterns and immune characteristics between CDS high- and low-risk groups

**FIGURE 5:**
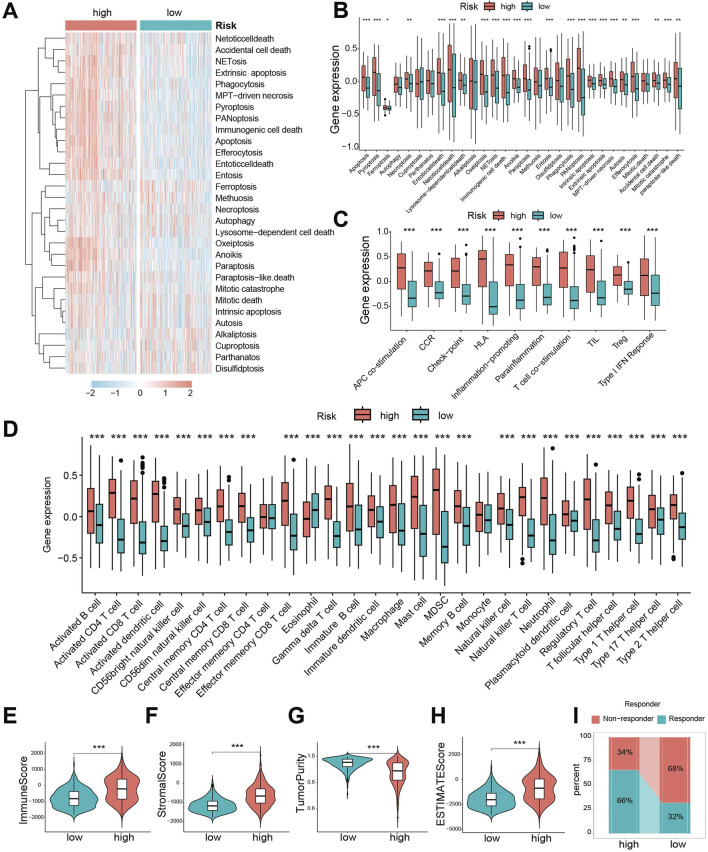
Significant differences exist in cell death patterns and immune characteristics between CDS high- and low-risk groups. **(A)** Heat map of 30 cell death score differences between CDS-high and CDS-low risk groups; **(B)** Box plot of 30 cell death score differences between CDS high and low risk groups; **(C)** ten kinds of immune function score difference boxplot. CDS (Cell-Death Score) **(D)** Box plot of difference in abundance of 28 immune cells between CDS-high and CDS-low risk groups; **(E)** Violin plot of Immune Score difference between CDS high and low risk group; **(F)** CDS high risk group of patients Score matrix (Stromal Score) differences violin figure; **(G)** the CDS Purity of high and low risk group of patients with Tumor (Tumor Purity) differences violin figure; **(H)** CDS high risk group of patients ESTIMATE score differences violin figure; **(I)** Bar plot of predicted percentage of TIDE immunotherapy for patients in CDS high and low risk groups. CDS (Cell-Death Score). *** indicates P < 0.001.

To compare the 30 cell death modalities between risk groups, we calculated death scores for each patient. Heatmaps visualization indicated higher score across most cell death modes in the high-risk group (Figure 5A). Specifically, 23 death modes showed significantly elevated scores in high-risk patients (Figure 5B). Similarly, immune function scores were significantly increased in the high-risk group (Figure 5C).

Given the crucial role of immune cells in glioma, we evaluated immune infiltration using single-sample gene set enrichment analysis (ssGSEA). The high-risk group exhibited significantly greater abundance across 28 immune cell types (Figure 5D), confirming enhanced immune infiltration. Consistently, Immune Score (Figure 5E) and Stromal Score (Figure 5F) were significantly higher, while Tumor Purity (Figure 5G) was lower, in the high-risk group. Consequently, the ESTIMATE scores were significantly elevated in high-risk patients (Figure 5H), suggesting they might be better candidates for immunotherapy. Assessment using the Tumor Immune Dysfunction and Exclusion (TIDE) algorithm predicted a better response to immunotherapy in the low-risk group (Figure 5I).

### CDS association with drug sensitivity

**FIGURE 6:**
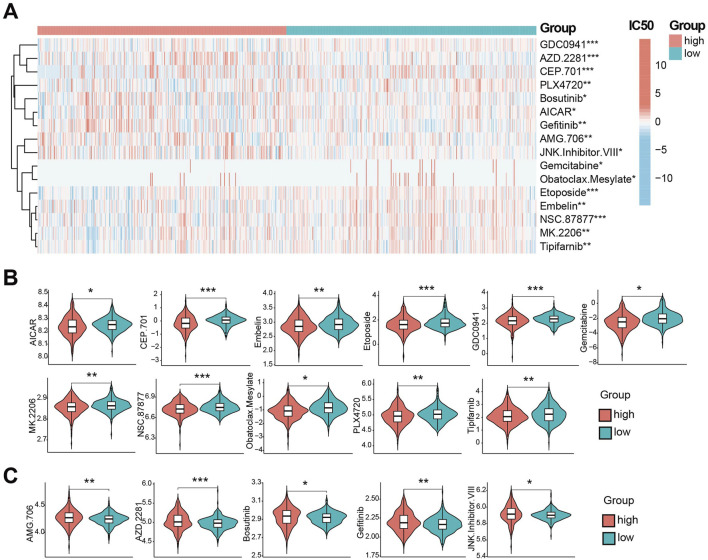
CDS association with drug sensitivity. **(A)** the CDS between high and low risk groups IC50 half inhibitory concentration level heat 16 kinds of drugs. **(B)** Violin plot of IC50 lower levels of CDS high-risk groups; **(C)** Violin plot of IC50 lower levels of CDS low-risk groups; * indicates P < 0.05, ** indicates P < 0.01, *** indicates P < 0.001.

Using GDSC database, we predicted drug susceptibility differences between risk groups. Significant differences in the half maximal inhibitory concentration, half inhibitory concentration (IC50) were observed for 16 drugs (Figure 6A). Eleven drugs showed lower IC50 (indicating higher sensitivity) in the high-risk group (Figure 6B): AICAR, CEP.701, Embelin, Etoposide, GDC0941, Gemcitabine, MK. 2206, NSC.87877, Obatoclax. Mesylate, PLX4720 and Tipifarnib. Conversely, five drugs showed lower IC50 (higher sensitivity) in low-risk group (Figure 6C): AMG.706, AZD.228, Bosutinib, Gefitinib and JNK. inhibitor.VIII. These represent potential therapeutic agents differential efficacy based on CDS risk stratification.

### Construction and validation of a nomogram integrating CDS and clinical features

**FIGURE 7:**
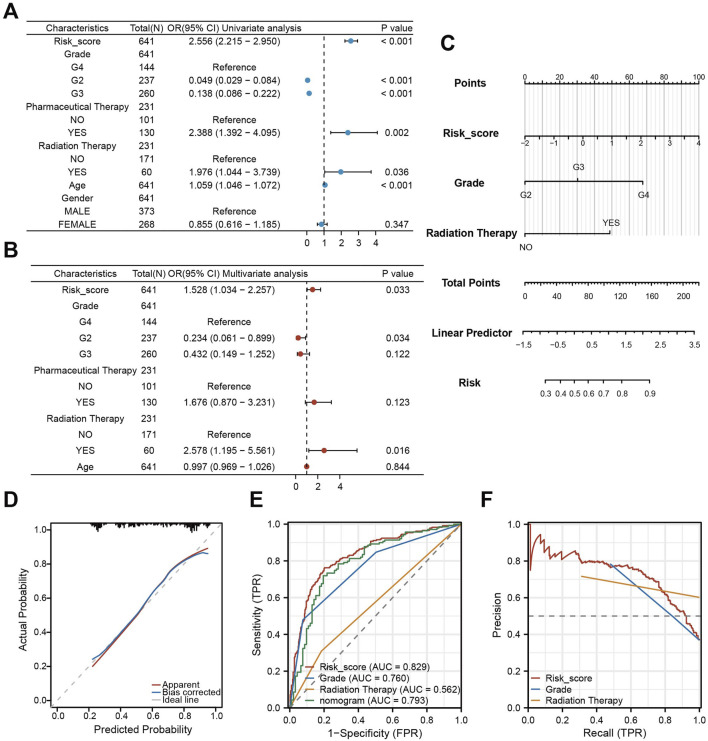
Construction and validation of a nomogram integrating CDS and clinical features. **(A)** Forest plot of the results of univariate COX regression analysis of CDS score and other clinical factors in the prognosis of patients; **(B)** Forest plot of CDS score and key clinical factors in patients’ prognosis by multivariate COX regression analysis; **(C)** CDS score combined with World Health Organization (WHO) grade and clinical model of radiotherapy Nomogram; **(D)** nomogram model fitting curve; **(E)** Receiver Operating Characteristic curve (ROC) curves of CDS score, World Health Organization (WHO) grade, radiotherapy and nomogram score; **(F)** PR (Precision-Recall) curve of CDS score, World Health Organization (WHO) grade and radiotherapy.

To evaluate the combined prognostic power of CDS with clinical factors, we conducted univariate COX regression analysis. This analysis identified CDS risk score, WHO grade, chemotherapy, radiotherapy, and clinical features as significant prognostic factors (Figure 7A). Subsequent multivariate COX regression confirmed CDS risk score, WHO grade, and radiation therapy as independent prognostic predictors (Figure 7B). Therefore, we integrated these three factors into a prognostic nomogram (Figure 7C). The calibration curve indicated good agreement between predicted and observed outcomes (Figure 7D). ROC curve analysis comparing the nomogram, CDS alone, WHO grade, and radiotherapy showed the highest AUC for CDS (0.829), followed by the nomogram (0.793) (Figure 7E). Precision-recall (PR) curve analysis further confirmed strong performance of the CDS risk score (Figure 7F). Notably, adding clinical factors to CDS did not improve prognostic performance beyond CDS alone.

### Single-cell level analysis of CDS

**FIGURE 8:**
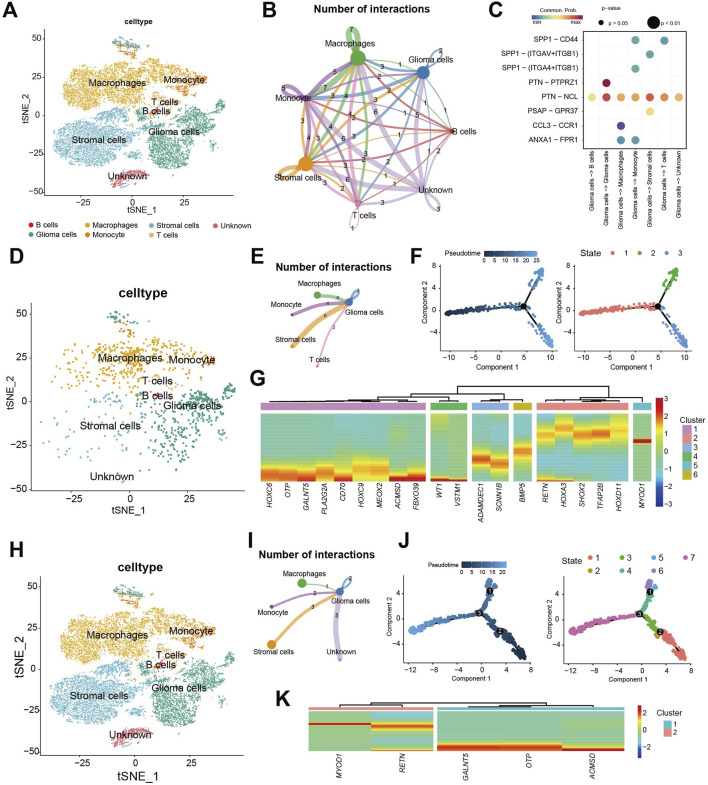
Single-cell level analysis of CDS. **(A)** Using 22,732 cells Seurat t distribution stochastic neighborhood embedded (t - SNE) cell type annotation figure; **(B)** by analyzing cell communication between main 6 types of cell cell communication situation of the network diagram; **(C)** Ligand-receptor interaction pairs for communication between glioma cells and other cell types. **(D)** Seurat t-distributed random neighborhood embedding (t-SNE) plot using 1134 CDS high-risk cells; **(E)** CDS high-risk cell populations and other types of cell communication network diagram; **(F)** CDS high-risk cell populations pseudo-time trajectory analysis, trajectories are colored from dark blue to light blue according to gradient; CDS cell developmental state trajectory of high-risk cell population; **(G)** 20/25 model gene expression under the false time trajectory heat maps of high-risk cells. **(H)** Seurat t-distributed random neighborhood embedding (t-SNE) plot using 21,598 CDS low-risk cells; **(I)** CDS low-risk cell populations and other types of cell communication network diagram; **(J)** CDS low-risk cell populations pseudo-time trajectory analysis, trajectories are colored from dark blue to light blue according to gradient; CDS cell developmental state trajectory of low-risk cell population; **(K)** 5/25 model gene expression under the false time trajectory heat maps of low-risk cells.

We further analyzed CDS using the GSE167960 single-cell RNA-seq dataset from 6 HGG patients (22,732 TME cells after quality control, Supplementary Figure S1). Manual annotation identified major cell types: glioma cells, monocyte, macrophages, stromal cells, T cells, and B cells [Figure 8A (Supplementary Figures S2A,B)], with proportions varying per patient (Supplementary Figure S2C). Assigning CDS risk at the single-cell level divided cells into high-risk (1,134 cells) and low-risk (21,598) groups, with the majority being low-risk (Supplementary Figure S2D). Cell communication analysis showed diverse interactions between these cell types (Figure 8B), including specific receptor-ligand interaction involving gliomas cells (Figure 8C).

Analysis of the high-risk cell populations showed its distribution across annotated cell types (Figure 8D). Cell communication analysis highlighted interactions, particularly between glioma cells and stromal cells, monocytes, macrophages, and T cells (Figure 8E). Pseudotime trajectory analysis of high-risk cell revealed a developmental path with one branch point, resulting in three distinct cellular states (Figure 8F). Expression analysis along the trajectory showed significant changes for 20 of the 25 CDS model genes, suggesting their key roles in the development of these high-risk cells (Figure 8G). High-risk cells exhibited 3.2× more interactions than low-risk cells (p < 0.001).

Analysis of the low-risk cell populations similarly showed its distribution (Figure 8H) cell communication patterns (Figure 8I). After, we have a group of CDS low-risk cells cells to time series analysis. Pseudotime trajectory analysis of low-risk cells revealed a path with three branch points, partitioning cells into seven distinct states (Figure 8J). Among the 25 CDS model genes, 5 showed significant dynamic expression changes during low-risk cell development (Figure 8K).

In summary, comparison of cell communication and pseudo-time trajectories between CDS high- and low-risk cell populations revealed differences. Gliomas cells within the high-risk population exhibited more intensive communication, both amongst themselves and with other cell types. Furthermore, a greater number of model genes showed significant expression changes during the developmental trajectory of the high-risk cell population.

### Experimental validation of 25 key genes i*n vitro*

**FIGURE 9:**
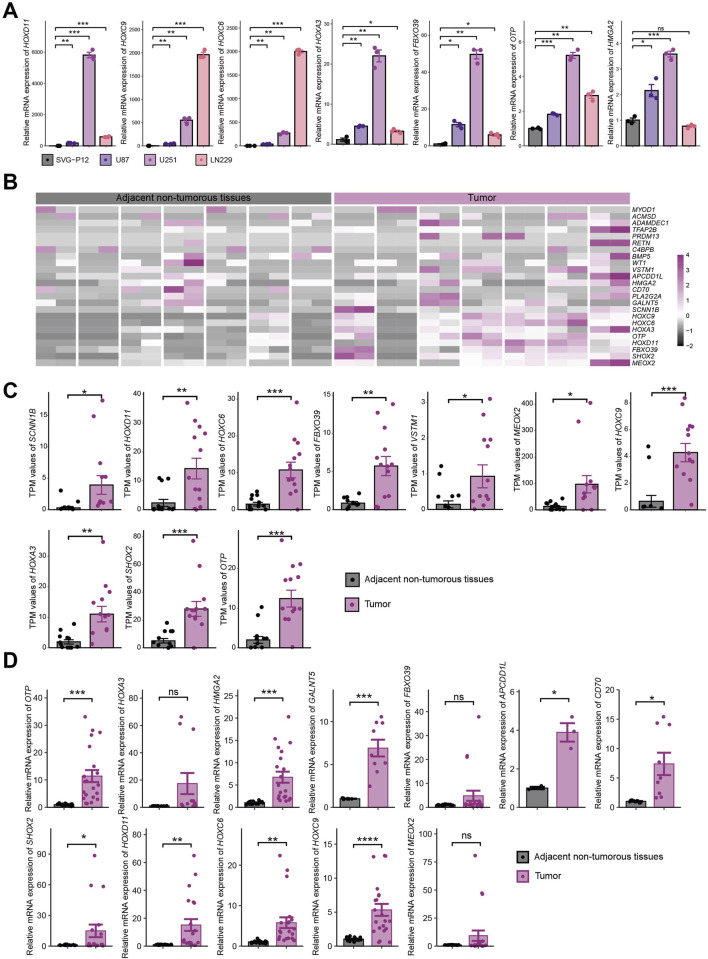
Experimental Validation of 25 Key Genes *In Vitro*. **(A)** mRNA expression of 7 key genes consistently detectable in normal astrocytes (SVG-P12) and tumor cell lines (U87, U251, LN229); **(B)** RNA-seq expression heatmap of key genes in 7 paired tissue samples; **(C)** Statistically significant expression of key genes in RNA-seq analysis; **(D)** Expression of 12 genes within normal threshold range by qRT-PCR across 7 paired samples. * indicates P < 0.05, ** indicates P < 0.01, *** indicates P < 0.001, **** indicates P < 0.0001.

Based on the above results, we performed mRNA-level detection of the 25 key genes *in vitro*. In cell line experiments, certain genes were undetectable due to low expression levels. Among the seven key genes (*HOXD11*, *HOXC9*, *HOXC6*, *HOXA3*, *FBXO39*, *OTP*, and *HMGA2*) consistently detectable in both normal astrocyte cell lines (SVG-P12) and glioma cell lines (U87, U251, LN229), tumor cell lines exhibited significantly upregulated expression compared to normal cell lines (Figure 9A). RNA-seq analysis of seven paired gliomas and adjacent non-tumorous tissue samples from our institution showed that 24/25 key genes were detectable, with the majority highly expressed in tumor samples (Figure 9B). Among these, 10 genes (*SCNN1B*, *HOXD11*, *HOXC6*, *FBXO39*, *VSTM1*, *MEOX2*, *HOXC9*, *HOXA3*, *SHOX2*, *OTP*) exhibited statistically significant differential expression (Figure 9C), while 14 genes showed non-significant differences (Supplementary Figure S3A). qRT-PCR analysis of these seven paired samples revealed undetectable expression for partial genes due to low expression levels. Among the 12 genes (*APCDD1L*, *CD70*, *FBXO39*, *GALNT5*, *HMGA2*, *HOXA3*, *HOXC6*, *HOXC9*, *HOXD11*, *SHOX2*, *MEOX2*, *OTP*) within normal detection thresholds, differential expression was observed between tumor and adjacent tissues (Supplementary Figure S3B). Integration of qRT-PCR data from all seven sample pairs demonstrated statistically significant expression differences for nine genes, while the remaining three genes showed higher mean expression in tumor tissues than in adjacent tissues but lacked statistical significance due to substantial dispersion (Figure 9D).

### Spatial transcriptomic analysis of 25 key genes

**FIGURE 10:**
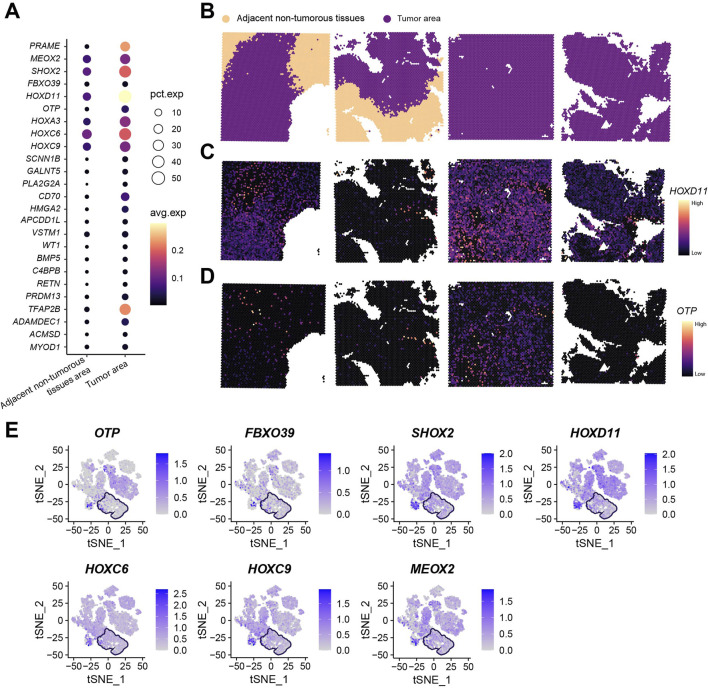
Spatial Transcriptomic Analysis of 25 Key Genes. **(A)** Total expression levels and average expression levels of 25 key genes in tumor regions *versus* adjacent non-tumorous areas across four spatial transcriptomic samples; **(B)** Spatial mapping profiles of four spatial transcriptomic samples; **(C)** Spatial mapping of *HOXD11* gene; **(D)** Spatial mapping of *OTP* gene; **(E)** Expression profiles of *OTP*, *FBXO39*, *SHOX2*, *HOXD11*, *HOXC6*, *HOXC9*, and *MEOX2* (boxed areas indicate adjacent non-tumorous tissue regions).

Our research group previously selected four surgically resected glioma specimens (including two paired tumor-adjacent tissues and two tumor-only tissues) for spatial transcriptomic analysis using the standardized Seurat analytical pipeline (Yang et al., 2024). Following Harmony integration of the four samples, dimension reduction and clustering yielded 17 transcriptionally distinct cell clusters (Supplementary Figure S4B,C). Given the established correlation between malignant transformation and large-scale chromosomal aberrations, inferCNV was employed for copy number variation (CNV) profiling. Consistent with prior findings, clusters 9 and 13 were designated as adjacent non-tumorous reference populations (Supplementary Figure S4D). Analysis revealed that cluster 3 additionally exhibited the genomic stability characteristic of adjacent non-tumorous tissues. Spatial transcriptomics demonstrated universal upregulation of the 25 key genes within tumor regions (Figure 10A; Supplementary Figure S4E). Spatial visualization confirmed distinct anatomical boundaries between tumor and adjacent non-tumorous zones (Figure 10B). Localization analysis of *HOXD11* and *OTP*—selected for high expression abundance and consistency with *in vitro* validation—revealed predominant tumor-specific localization in paired tumor-adjacent samples (*n* = 2), while showing diffuse distribution in tumor-only samples (*n* = 2) (Figures 10C,D). Integration of seven key genes consistently overexpressed in both tumor cell lines and tissues further revealed their tumor region-specific enrichment (Figure 10E; boxed areas indicate adjacent non-tumorous tissues).

## Discussion

Glioma heterogeneity necessitates deciphering programmed cell death (PCD) networks to overcome therapeutic resistance (Nicholson and Fine, 2021). Our integrated analysis of 30 PCD modalities transcends single-pathway limitations, revealing how coordinated cell death regulation drives glioma progression. The machine learning-derived Cell-Death Score (CDS) robustly stratifies patients, with high-risk groups exhibiting elevated genomic instability (TP53-dominant mutations, increased TMB/Mutation Count), immune checkpoint activation (PD-L1/CTLA-4), and distinct microenvironment remodeling (Lin et al., 2024; Gong et al., 2018; Liang et al., 2024). Crucially, high-CDScore patients show enhanced sensitivity to 11 agents (gemcitabine, etoposide) while displaying immune-activation signatures suggesting immunotherapy candidacy (Rajkomar et al., 2019; Greener et al., 2022). This discovery indicates that integrating the CDS model may uncover additional molecular markers and therapeutic targets.

Single-cell resolution confirmed developmental heterogeneity: High-risk cells progressed through trajectories dynamically regulated by 20/25 CDS genes, while differential intercellular communication involved oncogenic pathways (SPP1-CD44, HIFα-VEGF) (Sabu et al., 2023; Suvà and Tirosh, 2020; Fan et al., 2024; Xing et al., 2023; Chen et al., 2019; Tu et al., 2022). Key regulators include HOX family members (*HOXC9/C6/D11*) driving immunosuppression and invasion (Wang et al., 2021; Li et al., 2018; Liu et al., 2024b), *MEOX2* maintaining stemness via ERK/MAPK (Tachon et al., 2021; Schönrock et al., 2022; Li et al., 2024), and *GALNT5* mediating chemoresistance through DNA damage repair mechanisms (Jia et al., 2024). *CD70* overexpression further represents a therapeutically targetable axis of immune evasion (Junker et al., 2005; Wischhusen et al., 2002).

The marginal AUC reduction in the nomogram likely reflects information redundancy between CDS and clinical variables, where CDS encapsulates molecular features beyond WHO grade or treatment history. Among the 25 key genes, 13 were undetectable by qRT-PCR in some cell lines/tissues due to expression levels below technical detection thresholds (≤10 copies/ng RNA under 10 ng input and Ct cutoff = 35 cycles). RNA-seq analysis confirmed detectable expression of 24/25 genes (FPKM≥0.1), suggesting biological relevance despite qRT-PCR limitations. Spatial transcriptomics further validated tumor-specific expression of all genes.

Although contemporary glioma clinical practice relies on multiple established biomarkers—including MGMT promoter methylation status, IDH, genetic alterations beyond 1p/19q and adjuvant therapies—these markers primarily focus on single therapeutic contexts or specific pathological subtypes. As the understanding of glioma biology continues to expand, researchers are investigating emerging prognostic factors and novel molecular markers to refine prognostication and personalize treatment approaches. In this study, the differential enrichment of TP53 and IDH1 mutations in CDS high/low groups, which was independent of known molecular subtypes, further substantiates this perspective, this model can circumvent the limitations of single-molecular subtyping to predict patient prognosis. The CDS prognostic model constructed herein achieves cross-molecular subtype survival prediction in glioma for the first time by integrating 30 programmed cell death (PCD) modalities. This integration enables CDS to overcome the constraints of traditional biomarkers, making the development of combination therapeutic strategies targeting PCD pathways a novel tool for glioma treatment.

These findings establish CDS as a multidimensional biomarker integrating PCD biology with clinically actionable insights. The model’s prognostic power persists after controlling for WHO grade/radiotherapy, and its risk-specific drug sensitivity profiles enable personalized therapeutic selection. Mechanistically, the dysregulated expression of CDS components highlights novel targets for modulating glioma progression (Wu et al., 2024; Leung et al., 2002; Qian et al., 2023).

This study has several limitations: the cohort size for single-cell heterogeneity analysis is constrained, statistical power is compromised by the limited spatial transcriptomics sample size, and computational predictions of drug responses require validation via PDX/organoid models. Future investigations should expand sample cohorts and integrate dynamic metabolic profiling to better elucidate interactions between programmed cell death (PCD) and the tumor microenvironment (TME), thereby facilitating the development of combination strategies to overcome therapeutic bottlenecks in glioma.

## Conclusion

This study establishes the Cell-Death Score (CDS), a clinically translatable prognostic biomarker derived from machine learning integration of 30 programmed cell death (PCD) modalities. By dividing glioma patients into high- and low-risk groups and characterizing cellular subpopulations, the CDS framework reveals the mechanistic nexus between PCD heterogeneity, immune dysregulation, and therapeutic resistance. These insights provide novel molecular targets and actionable therapeutic strategies, enhancing our understanding of PCD-driven immune microenvironment remodeling. Future research should focus on deciphering dynamic PCD regulatory networks to optimize personalized therapeutic regimens and improve clinical outcomes.

## Materials and methods

### Data download

To develop a glioma prediction model based on the origin of homologous recombination deficiency for clinical precision medicine in Gliomas, this study acquired the gliomas dataset TCGA-GBM/LGG from UCSC Xena (Goldman et al., 2020) (https://xena.ucsc.edu). Download Count and sequencing of gene expression data in patients with FPKM values (n = 1,131), and further standardized into TPM value. At the same time, the clinical data of patients, including age, gender, survival time and survival status, were downloaded, and the patients without clinical information were excluded. At the same time, the Mutation data of patients were downloaded through GDC, and “Masked Somatic Mutation” was selected, visualized using maftools (Mayakonda et al., 2018) R package, and the tumor mutation burden (TMB) of each patient was obtained. Fraction Genome Altered (FGA, part of the Genome change scores), Mutation Count (mutations) and MSI - Sensor score obtained from cBioPortal database (http://www.cbioportal.org), Finally, a total of 641 samples meeting the criteria were retained. The normal human brain tissue gene expression dataset TcgaTargetGTEx (n = 1,664) was downloaded from the GTEx database, and the data type was selected as FPKM and converted to TPM format. Finally, a total of 1,141 normal human brain tissue gene expression data were obtained. From a GEO database (Barrett et al., 2013) (https://www.ncbi.nlm.nih.gov/geo) to download patients with Gliomas RNAseq data sets: GSE108474 (Gusev et al., 2018) (*Homo sapiens*, GPL570, a total of 550 patient tumor samples), which were all confirmed solid tumor samples of Gliomas patients; Download the single-cell expression profiling dataset at the same time: GSE167960 (Wang et al., 2023) (*H. sapiens*, GPL20301, tumor samples from 6 patients), single cell data were processed by Seurat package, and a total of 22,732 cells were obtained after quality control to filter out low-quality cells.

Cell communication between cell subsets was analyzed by CellChat package. The gene expression data of brain Gliomas dataset and clinical information of patients (including survival time and survival status) were downloaded from CGGA (Chinese Glioma Genome Atlas) database (Zhao et al., 2021) (http://www.cgga.org.cn/). The data samples were obtained from *H. sapiens*. All patients pathologically diagnosed with Gliomas were selected, and samples of patients lacking clinical staging information and survival information were excluded. Finally, two Gliomas patient datasets CGGA_693 (Zh et al., 2022) and CGGA_325 (Zhao et al., 2017). Were retained, and a total of 970 tumor samples were included in this study (Supplementary Table S1).

### Collection of 30 genes related to cell death modes

We conducted literature retrieval and based on previous literature reports (Tang et al., 2019; Galluzzi et al., 2018; Qin et al., 2023; Zou et al., 2022) and GeneCards database (https://www.genecards.org/) were collected 30 kinds of PCD model and the key regulatory genes, Including 228 genes related to apoptosis, 52 genes related to Pyroptosis, 753 genes related to Ferroptosis, 232 genes associated with Autophagy, necrotizing apoptosis (Necroptosis) phase There were 67 genes related to apoptosis, 12 genes related to Cuproptosis, 23 genes related to PARP-1-dependent cell death, 9 genes related to Entotic cell death. Three genes related to Netotic cell death, 240 genes related to Lysosome-dependent cell death, 3 genes related to Alkaliptosis, 3 genes related to cuproptosis. 19 genes related to Oxygen death (Oxeiptosis), 69 genes related to neutrophils inflammatory cell death (NETosis), 34 genes related to immunogenicity (Immunogenic_cell_death), cell death loss nest apoptosis related gene 434 (up), 29 genes related to Paraptosis, 8 genes related to Methuosis, 23 genes related to cell invasive death, 10 genes related to Disulfidptosis, 29 genes related to Phagocytosis, 19 genes related to PANoptosis, 9,255 genes related to Intrinsic apoptosis, 2108 genes related to Extrinsic apoptosis, 156 genes related to Mitochondrial permeability transition (MPT-driven necrosis), 3,838 genes related to Autosis, 102 genes related to Efferocytosis, 8,097 genes related to Mitotic death, 540 genes related to Accidental cell death, 946 genes related to Mitotic catastrophe, 6 genes related to paraptosis like death, and a total of 11,681 genes related to programmed cell death (Supplementary Table S2).

### Determine the feature genes associated with cell death

We used Limma R package (Ritchie et al., 2015) to perform differential analysis on the expression data of normal human brain tissue and tumor samples of gliomas patients, screened differentially expressed genes, and selected log_2_fold change >1 and P < 0.05 as cutoff. The obtained log_2_foldchange greater than 1 was the highly expressed gene in gliomas patients, and the log_2_Foldchange less than −1 was the low-expressed gene in gliomas patients. The volcano plot was used to show the distribution of these genes. In addition, the number of 30 cell death patterns collected was visualized by petal diagram, and the Venn diagram was used to show its intersection with differential genes. Finally, 30 differentially expressed genes related to cell death were obtained.

The bar chart was used to visualize the composition of 30 cell death and non-cell death genes in the downregulated degs. To explore the biological significance of these differentially expressed Genes related to cell death, we used Gene Ontology (GO) (The Gene Ontology Consortium, 2017) and Kyoto Encyclopedia of Genes and Genomes (KEGG) (Chen et al., 2020) enrichment analysis was used to evaluate the signaling pathways and biological processes associated with the differentially expressed genes, with a Q-and P-value threshold of <0.05.

### Unsupervised clustering based on differential genes reveals differences in immune characteristics among subgroups

The “Consensus Cluster Plus” (Seiler et al., 2010) R package was used to identify multiple cell death-related subtypes through unsupervised consensus clustering, and the k range was 2–10. To ensure the stability of clustering, we repeated 1,000 times. Considering the feasibility of clinical prognostic analysis, the optimal number of clusters consists of two maximizing intra-cluster consensus while minimizing ambiguity in cluster assignment. CIBERSORT (https://cibersort.stanford.edu/) is based on linear support vector regression (linear support vector regression) subtype of principle of human immune cells to the expression of matrix convolution (Newman et al., 2019) It can evaluate the infiltration status of immune cells in sequencing samples based on the gene expression feature sets of 22 known immune cell subtypes. This study by CIBERSORT algorithm with different coronary heart disease (CHD) samples consolidated data sets to evaluate immune cells into the state, and then by Wilcoxon test different diseases in various immune cell infiltration of subgroup differences, P < 0.05 or less for the difference was statistically significant. Principal component analysis (PCA) was used to observe the differentiation between subgroups, and volcano plot was used to display the differences between subgroups to further screen the signature genes.

### Multi-machine learning to realize one-stop prognostic feature gene screening and prognostic model construction

In order to construct a stable prognostic model for gliomas based on multi-cell death mode, (1) first, we integrated 10 classical algorithms: Random forest (RSF), least absolute shrinkage and selection operator (LASSO), gradient boosting machine (GBM), Survival support vector machine (survival-SVM), supervised principal component (SuperPC), ridge regression (ridge), Cox Partial least squares regression (plsRcox), CoxBoost, Stepwise Cox, and elastic network (Enet). Among them, RSF, LASSO, CoxBoost, and Stepwise Cox have the function of dimensionality reduction and variable screening, and we combined them with other algorithms into 114 machine learning algorithm combinations. (2) Next, we used TCGA-GBM/LGG as the training cohort, and used the combination of these 114 algorithms to screen key genes and construct a prognostic model based on the previously identified feature genes. (3) Finally, in the three test cohorts (CGGA-693, CGGA-325, GSE108474), we used the key genes obtained in the training cohort to calculate the risk score for each cohort. According to the average C-index of the four test cohorts, we finally selected the best prognostic model and calculated its final risk Score, Cell-Death Score (CDS). Based on the median value of the score, the patients were divided into CDS high-risk group and CDS low-risk group. Survival analysis and receiver operating characteristic (ROC) curve analysis were used to evaluate the prognostic significance of CDS.

### Tumor mutation burden (TMB) and microsatellite instability (MSI) analysis

To analyze single nucleotide polymorphisms (SNPS) in different risk score subgroups of TCGA-GBM/LGG patients, maftools package was used to analyze frequently mutated genes in high and low risk groups. In the meantime, Through from cBioportal database (https://www.cbioportal.org) for patients with GBM TMB (Tumor Mutation Burden), MSI - Sensor Score, Fraction Genome Altered, Mutation Count, we analyzed the corresponding score differences between high and low-risk groups to reveal their mutation level characteristics. Meanwhile, the difference of Homologous recombination deficiency (HRD) score between high and low risk groups was analyzed.

### The comparison of cell death score and immune characteristics

In order to reveal the discriminative power of CDS risk score in tumor immunity, we performed single-sample gene set enrichment analysis (ssGSEA) (Foroutan et al., 2018) enrichment analysis. ssGSEA is a method used to assess the activity of gene sets (e.g., pathways or functional sets) in a single sample. It quantifies the degree of enrichment of gene sets by calculating the cumulative distribution function of genes within a sample, thereby revealing the activity associated with a specific biological process. By performing ssGSEA enrichment analysis of the related genes corresponding to the 30 cell death modes, we obtained the death score of each patient’s corresponding cell death mode, which was visualized by heat map and difference boxplot. At the same time, we obtained the scores of each patient in different immune functions and 28 immune cells in the same way. Next, we used the R package “estimate “to analyze the differences in tumor immune score, stromal score and tumor purity. Meanwhile, Tumor Immune Dysfunction and Rejection (TIDE) is a computational method that mimics the tumor immune escape mechanism and is used to evaluate the potential response to immune checkpoint blockade (ICB) treatment (Jiang et al., 2018) In website: http://tide.dfci.harvard.edu/. TIDE prediction was performed on youdaoplaceholder0, and the percentage difference of immunotherapy response prediction results between high and low risk groups was analyzed.

### Development and validation of potential therapeutic drugs

In order to assess the CDS drug sensitivity difference between high and low risk group of patients, we based on anti-cancer drug sensitivity genomics database (https://www.cancerrxgene.org/), Genomicsof Drug Sensitivity in Cancer (GDSC) was used for drug sensitivity analysis using pRRophetic package. The drugs with significant difference in half-inhibitory concentration between the high and low risk groups were identified. To screen potential anticancer drugs with better efficacy in patients with different risk groups. In this study, we further classified the 16 candidate drugs screened in Figure 6 into the following categories: conventional chemotherapeutic agents (e.g., Etoposide, Gemcitabine), targeted kinase inhibitors (e.g., Bosutinib, Gefitinib, PLX4720, Tipifarnib), PI3K/AKT/mTOR pathway inhibitors (e.g., GDC0941, MK-2206, Embelin), and other small-molecule inhibitors (e.g., Obatoclax Mesylate, NSC-87877, JNK Inhibitor VIII, AICAR, CEP-701, AMG-706, AZD-2281). Drug classifications were confirmed based on annotations from the GDSC database and established pharmacological literature. Regarding the IC50 difference threshold, the pRRophetic model outputs relative predicted ln (IC50) values between patient subgroups rather than absolute clinical *in vivo* drug concentrations. Therefore, we primarily determined differential drug sensitivity based on statistical significance (Wilcoxon test, BH-adjusted q < 0.05). To ensure biological relevance, we further required a predicted median ln (IC50) difference >0.25 between groups (approximately equivalent to a 28.3% difference on the original scale) to define potential clinical relevance.

### Construction and validation of a nomogram model integrating CDS and clinical features

After univariate and multivariate Cox regression analysis of CDS and other clinical features, we integrated all identified independent prognostic parameters and constructed a prognostic nomogram using the R package “rms”. Calibration plot, ROC curve and decision curve analysis (DCA) were used to evaluate the predictive ability of the nomogram.

### Analysis of single-cell sequencing data

Single-cell RNA sequencing (scRNA-seq) data were preprocessed and analyzed using the “Seurat “R package. The “NormalizeData “function of “Seurat” software package was used to normalize the scRNA-seq data, and the normalization method was set to “LogNormalize”. The normalized data were then converted into Seurat objects. The percentage of mitochondrial or ribosomal genes was calculated and low-quality cells were excluded to ensure quality control (QC). We excluded samples with a gene count of less than 200 or more than 3,000, and samples with a ribosomal RNA ratio of more than 20%. Then, by using Seurat package FindVariableFeatures, choose the variable characteristics of 3,000 genes as the most important, as the basis of standardized scRNA - seq data of each cell. In addition, we implement ScaleData and RunPCA function to get the number of principal components (PC) based on object Seurat.

We use “UMAP (Uniform Manifold Approximation and Projection)” dimensionality reduction to further summarize the principal components. Finally, using the annotation information of each class of cells supported by previous articles (Abdelfattah et al., 2022; Wen et al., 2023), the Idents and DimPlot functions were used to annotates and visualize the cells of the major cell types or subtypes. Then, we performed CellChat (intercellular communication) analysis. CellChat is used to analyze the intercellular communication of R packages, including human and mouse ligand/receptor interaction database, can according to the comments for different cell clusters scRNA - seq intercellular communication network data analysis. First, we used CellChat to evaluate the major signal inputs and outputs between all types of cell clusters using CellChatDB.human. We then used the netVisual_circle function to show the strength of the intercellular communication network from the target cell cluster to different cell clusters in all clusters.

Meanwhile, in order to study the relationship between the cell pseudo-time traces and the model genes, we adopted the Monocle R package to obtain the single-cell RNA data of all cell types. Highly variable genes were set according to the following filtering criteria: mean expression ≥0.1 and empirical value of dispersion ≥1* Dispersion fit. The DDRTree method was used for dimensionality reduction. We then used the “plot_pseudotime_heatmap” function to visualize the heatmap of model gene dynamic expression in the pseudo-time traces showing different TME cell types in HGG. Significant dynamic changes were defined as p-value <0.05 and |log_2_FC|>1.

### RNA extraction and real-time qPCR analysis

RNA was extracted from cell lines and tissues by using TRIzol (1 mL for 50–100 mg brain tissue or 2–5 × 106 cells), and quantified by Nanodrop. 20 μg of RNA was revised transcribed by Revert Aid First Strand cDNA Synthesis Kit (Thermo Scientific #K1622) or miRNA first Strand cDNA Synthesis Kit (by tailing A) (Vazyme #MR201). Quantitative PCR (qPCR) was run with cDNA input in a 20 μL reaction using 2 × SYBR Green PCR Master Mix. For analysis, the ΔΔCt method was used to calculate the relative fold gene expression of samples. The housekeeping gene GAPDH served as control for qPCR. The primers used in these experiments are shown in Supplementary Table S3.

### RNA-seq sample processing, library preparation, sequencing, and data analysis

After extracting RNA from human glioma tissues and adjacent non-tumorous tissues, RNA’s total amounts and integrity were assessed using the RNA Nano 6000 Assay Kit of the Bioanalyzer 2100 system (Agilent Technologies, CA, United States). For library preparation, 1.5 mg of total RNA (RIN R 6.8) was used with VAHTS Universal V8 RNA-seq Library Prep Kit for Illumina (Vazyme, NR605). After the library is qualified, the different libraries are pooled according to the effective concentration and the target amount of data off the machine, then sequenced by the Illumina NovaSeq 6,000. The end reading of 100-bp pairing is generated.

The FASTQ format files obtained from the Illumina platform are transformed into short reads (raw data). Sequence quality control is performed using Fastp, which removes reads containing adapters, reads with N bases, and low-quality reads. All downstream analyses are based on clean data of high quality. For alignment, STAR (Version 2.7.9a) aligns the clean reads to the Human reference: Obtained from UCSC (GRCh38/hg38). Uniquely mapped reads are used for subsequent analyses. RSEM (RNA-Seq by Expectation–Maximization Version 1.3.1) and DESeq2 (v1.42.0) were used to identify differentially expressed genes (DEGs). Genes are considered differentially expressed if the log2(FoldChange) is either > 1 or < −1, and the adjusted p-value (p-adjust) is < 0.05. For experimental validation of the 25 previously identified key genes, expression patterns were visualized through heatmaps generated by pheatmap (v1.0.12) and quantitative bar plots constructed with ggplot2 (v3.5.2).

### Spatial sequencing data analysis

We used Seurat (version 5.2.1) to perform data processing, integration, and clustering of spatial transcriptomics data. Spatial expression matrices were loaded and individual Seurat objects were created for each of the four samples. Sample identities were assigned to each object and subsequently merged into a single integrated dataset. Expression matrices were normalized using NormalizeData and scaled using ScaleData Variable features were identified using FindVariableFeatures with default parameters. Principal component analysis was performed using RunPCA. Then armony integration was applied for batch correction through HarmonyIntegration with principal components (dims = 1:30) as input. The resulting integrated embeddings were used for downstream unsupervised analysis: Shared nearest neighbor graph construction was performed FindNeighbors and clustering was implemented with resolution parameter 0.6. All analysis steps used default parameters except where explicitly specified.

Copy number variations (CNVs) in six tumor samples were inferred using inferCNV (v1.22.0). An inferCNV object was constructed with three inputs: 1) the raw expression matrix from the integrated spatial dataset, 2) Seurat-derived cluster annotations, and 3) a gene positional file. Clusters 9 and 13—identified as non-tumor regions in previous research—served as the reference group. Analysis parameters included: an expression cutoff of 1 to filter low-abundance genes, group-based cell clustering, noise reduction, and hidden Markov modeling for CNV state prediction.

### Statistical analysis

All statistical analyses were performed with R software (versions 4.3.1 and 3.6.0). Comparison of inter-group differences using Wilcoxon rank-sum test and t-test The Kruskal-Wallis test was used to evaluate differences between more than two groups. The Spearman correlation method is adopted for correlation analysis. P < 0.05 was considered as the threshold of statistical significance.

## Data Availability

The datasets presented in this study can be found in online repositories. The names of the repository/repositories and accession number(s) can be found in the article/Supplementary Material.

## Supplementary material

SUPPLEMENTARY FIGURE S1 Single-cell Quality Control. **(A)** RNA Feature Count, Count count, mitochondrial and red blood cell proportion box plot after quality control; **(B)** quality control before the RNA Feature count, mitochondria and scatterplot proportion of red blood cells; **(C)** RNA Feature count, mitochondrial and red blood cell proportion scatter plot after quality control.

SUPPLEMENTARY FIGURE S2 Manual Annotation of Cell types **(A)** annotation cell t distribution stochastic neighborhood before embedding (t - SNE) figure; **(B)** markers in various types of cells in different subgroup of bubble chart, bubble color shades, said the average subgroup cells express, said the subgroup size cells expressed in proportion. **(C)** all kinds of cells in different patient samples component percentage histogram; **(D)** t distribution random neighborhood embedding (t-SNE) plot of cell distribution in CDS high and low expression groups.

SUPPLEMENTARY FIGURE S3 Experimental Validation of 25 Key Genes In tumor. **(A)** Expression of 14 key genes with no statistically significant differences in RNA-seq analysis; **(B)** qRT-PCR results of 12 key genes within normal threshold range across seven paired samples.

SUPPLEMENTARY FIGURE S4 Spatial Transcriptomic Analysis of 25 Key Genes. **(A)** Four distinct sources of glioma samples; **(B)** Standardized Seurat analytical workflow. Harmony-integrated dimensionality reduction and clustering of four sample groups identified 17 transcriptionally distinct cell clusters; **(C)** Spatial mapping of clustering results; **(D)** Copy number variation (CNV) analysis results; **(E)** Expression profiles of 25 key genes across distinct regions.
