# Supplementary figures and images for: Integrated machine learning analysis of 30 cell death patterns identifies a novel prognostic signature in glioma
Source: Front Cell Dev Biol. 2025 Sep 19;13:1677290. doi: 10.3389/fcell.2025.1677290 (PMC12491174; doi:10.3389/fcell.2025.1677290)

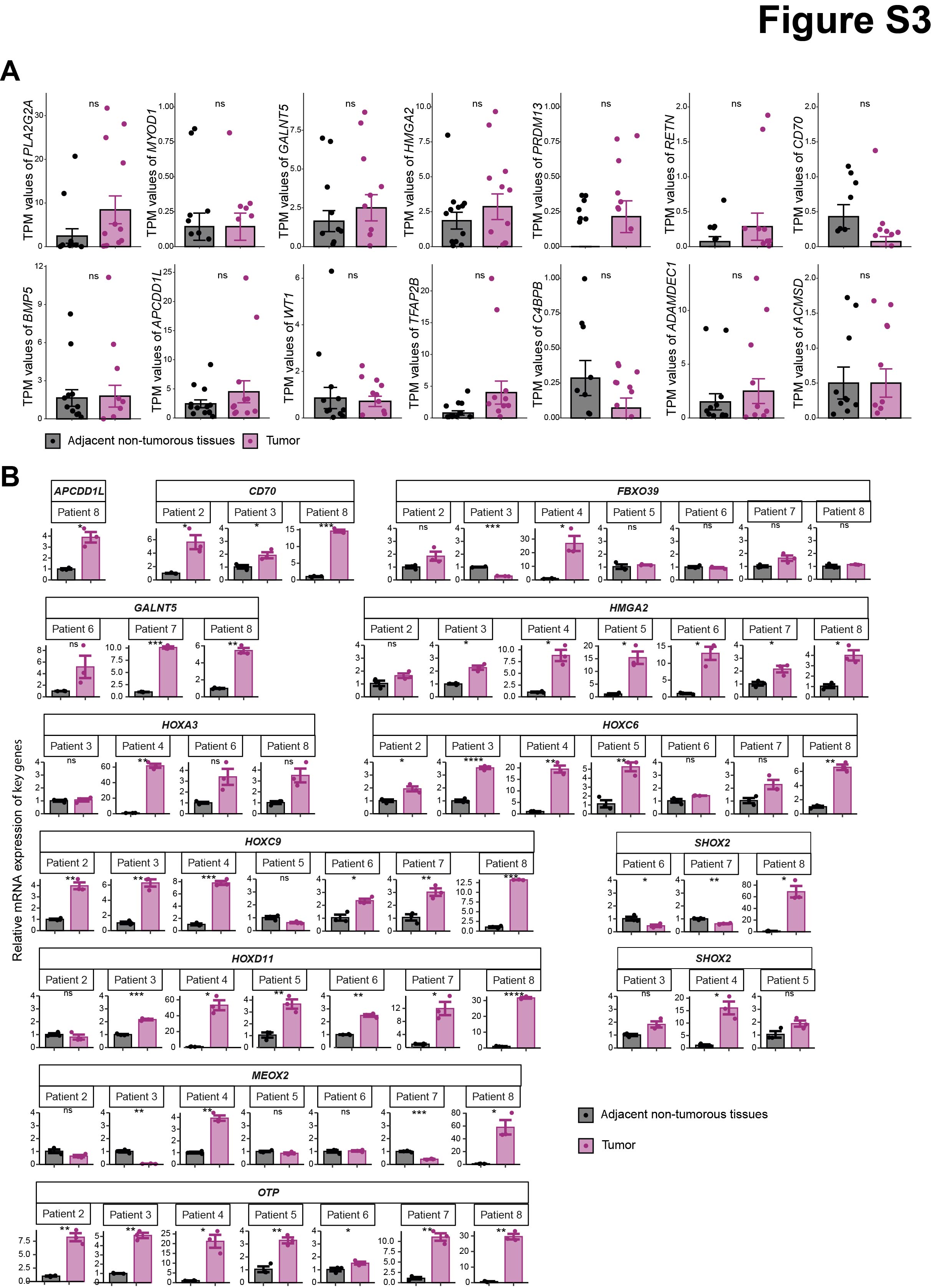

Supplement: Supplementary file 2 [file Image3.jpeg]

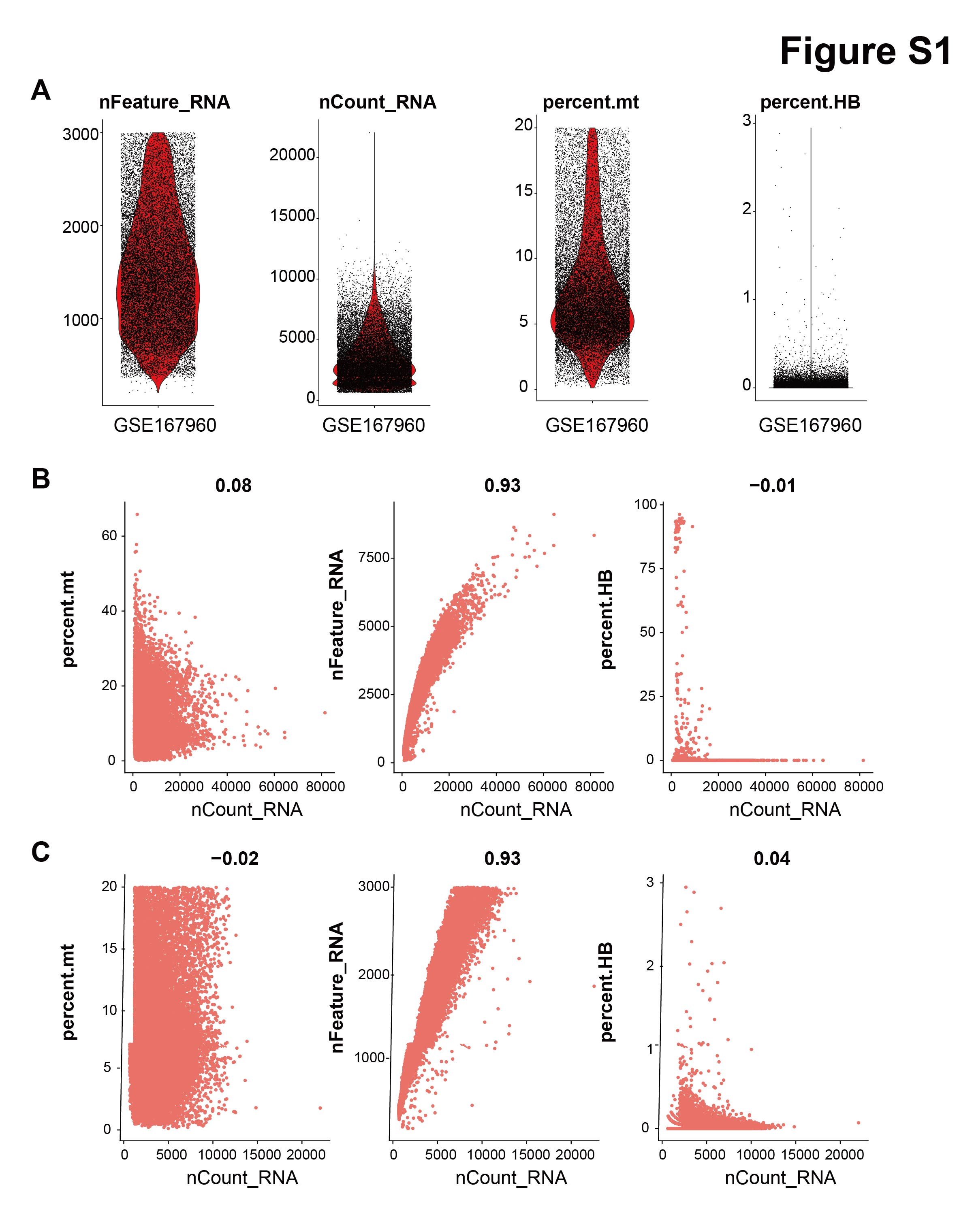

Supplement: Supplementary file 4 [file Image1.jpeg]

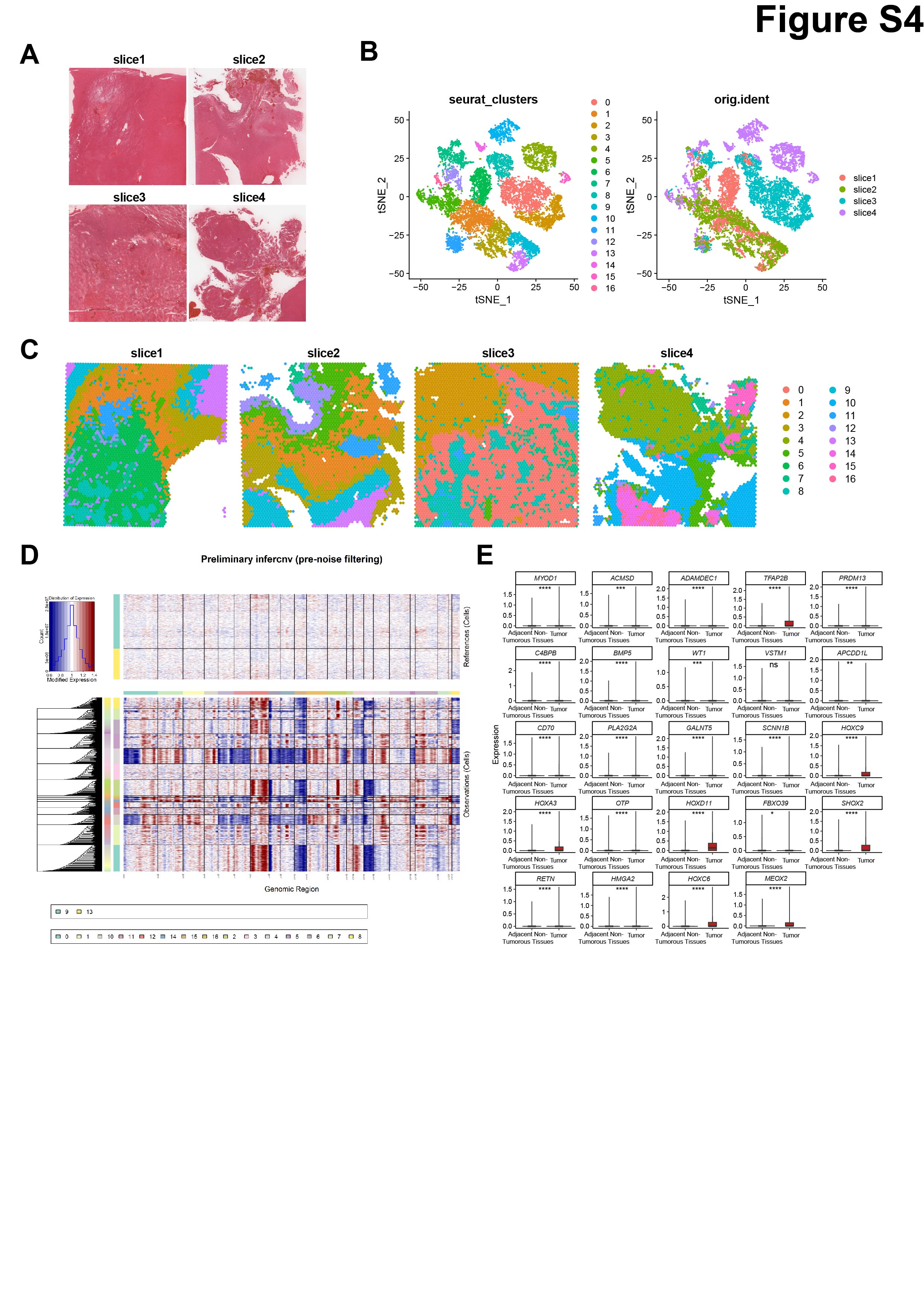

Supplement: Supplementary file 5 [file Image4.jpeg]

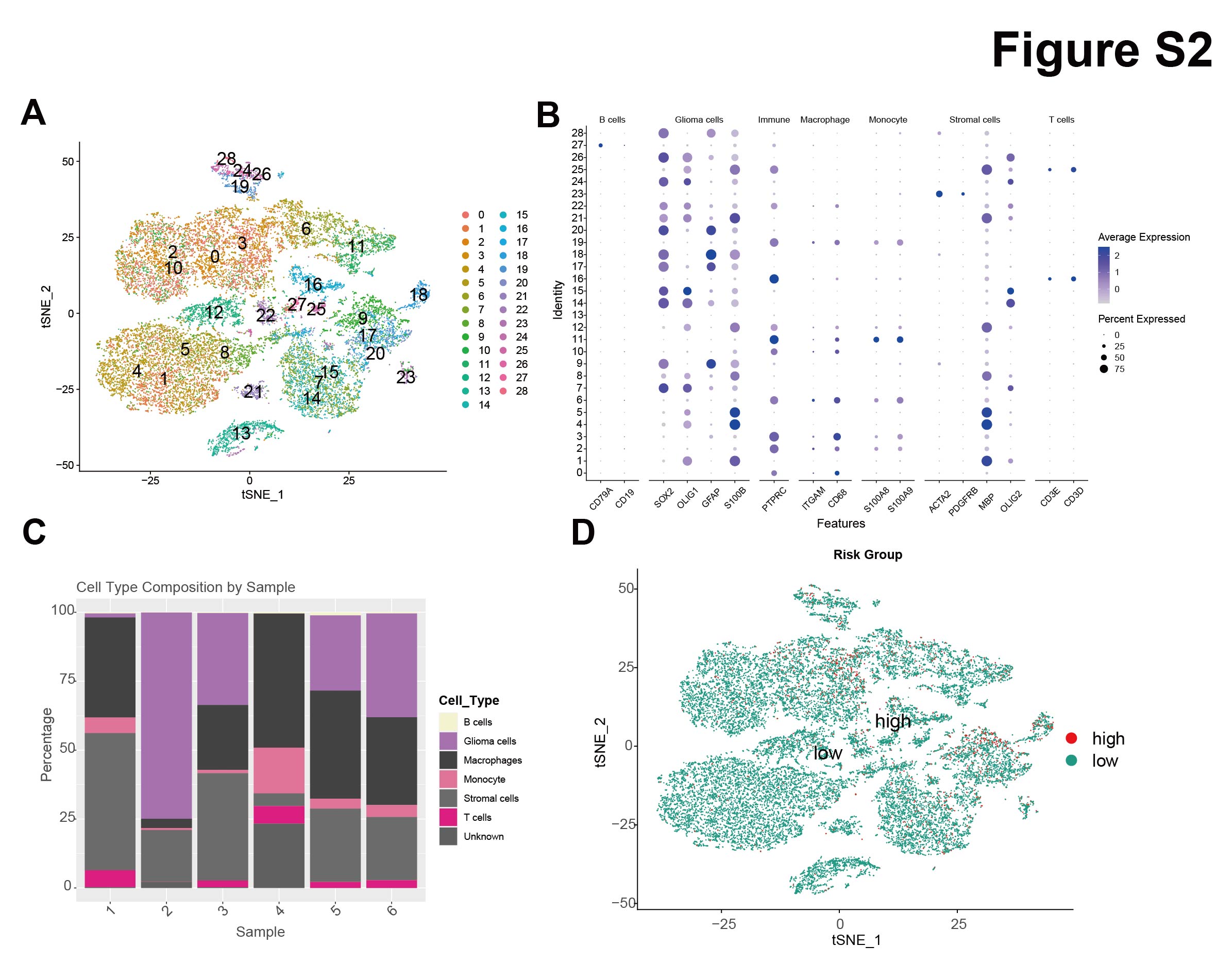

Supplement: Supplementary file 6 [file Image2.jpeg]
